# Changes to the mtDNA copy number during yeast culture growth

**DOI:** 10.1098/rsos.211842

**Published:** 2022-07-06

**Authors:** Ben Galeota-Sprung, Amy Fernandez, Paul Sniegowski

**Affiliations:** Department of Biology, University of Pennsylvania, Philadelphia, PA, USA

**Keywords:** mitochondria, mtDNA copy number, yeast, *S. cerevisiae*, mtDNA

## Abstract

We show that the mitochondrial DNA (mtDNA) copy number in growing cultures of the yeast *Saccharomyces cerevisiae* increases by a factor of up to 4, being lowest (approx. 10 per haploid genome) and stable during rapid fermentative growth, and highest at the end of the respiratory phase. When yeast are grown on glucose, the onset of the mtDNA copy number increase coincides with the early stages of the diauxic shift, and the increase continues through respiration. A lesser yet still substantial copy number increase occurs when yeast are grown on a nonfermentable carbon source, i.e. when there is no diauxic shift. The mtDNA copy number increase during and for some time after the diauxic shift is not driven by an increase in cell size. The copy number increase occurs in both haploid and diploid strains but is markedly attenuated in a diploid wild isolate that is a ready sporulator. Strain-to-strain differences in mtDNA copy number are least apparent in fermentation and most apparent in late respiration or stationary phase. While changes in mitochondrial morphology and function were previously known to accompany changes in physiological state, it had not been previously shown that the mtDNA copy number changes substantially over time in a clonal growing culture. The mtDNA copy number in yeast is therefore a highly dynamic phenotype.

## 1. Introduction

Mitochondria are membrane-enclosed organelles that originate from an ancient symbiosis [1,2]. Mitochondria enable a variety of capabilities, the most important of which is the production of ATP via aerobic respiration. To a varying degree across the eukaryotic tree of life, most originally mitochondrial genes have been assimilated into the nuclear genome [3], but in nearly all cases an independent mitochondrial genome (mtDNA) is maintained within the mitochondria. Generally, there are multiple copies of mtDNA per individual cell. In multicellular organisms, the copy number may vary substantially. For example, in humans, there are hundreds to tens of thousands of mtDNA copies per cell, with considerable variation between tissue type [4–7] as well as substantial inter-individual variation [8]. In humans, deficiency in mtDNA copy number is associated with a variety of human disease phenotypes [9–12].

The yeast *Saccharomyces cerevisiae* is an important model system for studying various aspects of mitochondrial biology [13]. The mitochondrial genome of *S. cerevisiae* encodes eight major proteins (the vast majority of the approx. 1000 mitochondrial proteins are encoded in the nuclear genome), of which seven are subunits of various respiratory enzyme complexes of the inner membrane [3]. The mtDNA copy number varies somewhat by strain [14]. An early study [15] found that 16–25% of all cellular DNA is mitochondrial; given an mtDNA genome of 75–85 kbp and a nuclear genome of approximately 12 Mbp, this suggests a copy number of approximately 25 to 50 per haploid genome, a range generally borne out by subsequent studies [16–18], though copy numbers as high as 200 have been observed in diploids [19]. Two recent studies have found approximately 18–20 mtDNA copies per haploid cell, and up to roughly threefold variability introduced by various gene deletions [20,21].

In yeast, as in other organisms, mtDNA is packed into protein–DNA complexes called nucleoids [18,22–24]. These contain mostly linear mtDNA in polydisperse (that is, not in units of discrete genomes) linear tandem arrays containing on average one to two copies of the mitochondrial genome [25,26], though under anaerobic conditions there may be many fewer nucleoids with many more mtDNA per nucleoid [27]. The mitochondria within the yeast cell form a highly dynamic network in which fusion and fission events occur very frequently [28,29]. There is generally a single ‘giant’ mitochondrion that is much bigger than the others, and the number of spatially distinct mitochondria varies with physiological state, being lowest in rapidly growing cells and highest in stationary phase cells [30].

*S. cerevisiae* that are grown with glucose as a carbon source characteristically ferment it into ethanol and then transition—the diauxic shift—into a fully respiring physiology upon the exhaustion of glucose supplies. The diauxic shift initiates a substantial reorganization of yeast metabolism in which expression patterns for different sets of proteins (e.g. those associated with oxidative phosphorylation, stress response, and the glyoxylate cycle) change dramatically in a coordinated and staged manner [31–33]. Much of this metabolic reorganization occurs at the mitochondrial level as the mitochondrial role transitions from biosynthetic hub to energy generator during the diauxic shift, which should probably be considered a distinct metabolic phase [34].

In this study, we investigate changes in mtDNA copy number over time by sampling repeatedly from growing cultures of *S. cerevisiae*, using haploid and diploid variants of both a common laboratory strain and a natural isolate. We find that the mtDNA copy number during fermentation is surprisingly low (approx. 10 per haploid genome). The onset of the diauxic shift is associated with the start of a large mtDNA copy number increase that, from rapid fermentative growth through to the stationary phase, is as high as fourfold or more. This suggests that the metabolic reorganization of the diauxic shift is associated with a substantial increase in mtDNA copy number. There is also a smaller mtDNA increase even when there is no diauxic shift (that is, when there is no fermentation phase). We confirm the reverse phenomenon of the copy number rise: when slowly growing cells are transferred to fresh media, the mtDNA copy number drops rapidly. We find that differences between the strains are most apparent after the fermentation phase. We find linear effects of ploidy, and find that the copy number increase has a complex relationship with cell size. These results suggest that the mtDNA copy number is a highly dynamic and plastic phenotype in yeast, and point to new directions for research into the regulation of mtDNA copy number in yeast.

## 2. Results

### 2.1. mtDNA copy number increases over time in growing cultures

**Figure 1.**
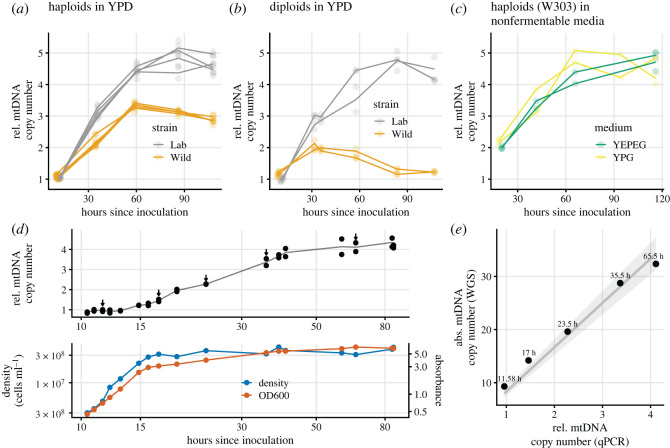
Relative mtDNA copy number over time in replicate cultures grown in rich media supplemented with glucose (*a*, haploids and *b*, diploids), or glycerol and glycerol + ethanol (*c*, W303 haploids only). DNA extractions were carried out for each culture at approximately 24-h intervals. Two replicate qPCR measurements were performed for each DNA extraction; the lines track the mean of the two measurements. The first extraction was always performed at OD600 = 0.5 (approx. 11 h after inoculation in YPD), which corresponded to approximately 3 × 10^7^ cells ml^−1^ in haploid strains and approximately 2.4 × 10^7^ cells ml^−1^ in diploid strains. (*d*) Detailed mtDNA copy number dynamics of a single haploid W303 culture grown in YPD, with time shown on a log scale to better visualize the earlier data points. For 5 of 17 timepoints, marked by arrows, the measurement of mtDNA copy number by qPCR was validated by a whole genome-sequencing (WGS) read-depth assay (*e*).

In our first experiment, we grew replicate cultures in rich liquid media supplemented with glucose (YPD). We employed haploid and diploid strains constructed from both a standard laboratory background (W303) and a natural woodland isolate. For all cultures, the first DNA extraction was performed at an absorbance corresponding to rapid fermentative growth (OD600 = 0.5), with subsequent extractions taking place at approximately 24 h intervals. The relative ratio of mtDNA to nuclear DNA (mt/nDNA) was assayed by qPCR. We found (figure 1*a*,*b*) that the mt/nDNA ratio increased approximately threefold for wild haploids, and over fourfold for both haploid and diploid laboratory strains. The mt/nDNA ratio per haploid genome was similar across the two different ploidies, consistent with prior results [14]. The exception was wild diploids, for which the mtDNA copy number first doubled but subsequently declined, likely because of their strong propensity to sporulate spontaneously under these conditions (laboratory strains tend to sporulate only if specifically induced).

### 2.2. mtDNA copy number estimation by WGS

Having established the general fact of mtDNA copy number increase for a variety of strains and conditions, we focused on the haploid laboratory strain for further study. We next sampled more extensively from a single culture grown on glucose, assaying the mt/nDNA ratio by qPCR at a total of 17 timepoints. These results demonstrated that the mt/nDNA ratio was stable during fermentation prior to the slowdown of growth associated with glucose exhaustion (figure 1*d*, early time points). As a control to rule out any effect of total time in culture, we also confirmed that the relative mtDNA copy number remains at approximately 1 when a culture is repeatedly transferred while still in fermentation for over 60 h (electronic supplementary material, figure S6).

For 5 of those same 17 timepoints for which qPCR assays were performed, we carried out a WGS read-depth assay, the purpose of which was to verify the qPCR results as well as to estimate the absolute (rather than relative) mtDNA copy number. We found that the absolute mtDNA copy number during fermentative growth for haploids is approximately 9, eventually increasing to over 30, and that there is good agreement (figure 1*e*) between the qPCR and WGS assays as to the scale of the increase in copy number, though the WGS assay shows a slightly smaller increase (electronic supplementary material, figure S1). As controls for the WGS assay, we assayed the copy number of various other repetitive genomic elements, which did not change over time (electronic supplementary material, figure S2). We also found good agreement between short- and long-read WGS-based assays for mtDNA copy number (electronic supplementary material, figures S3–S5).

### 2.3. mtDNA copy number increase and the diauxic shift

*S. cerevisiae* is a Crabtree-positive yeast: it ferments glucose into ethanol even when oxygen is present, and then approaching glucose exhaustion enters the diauxic shift, transitioning from fermentation of glucose to oxidative respiration of ethanol. We were interested in the association of the diauxic shift with the observed rise in mtDNA copy number. Therefore, we conducted another dense-sampling experiment in which ethanol and glucose concentrations were regularly measured. We also investigated changes in cell size in this experiment.

**Figure 2.**
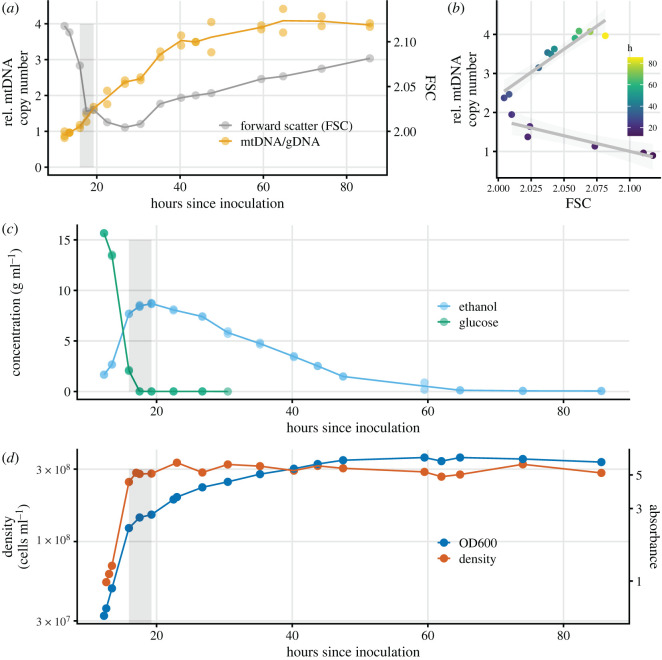
For a haploid W303 culture grown in YPD, measurements of various quantities over time. In *a*, *c*, and *d*, the grey rectangles show the approximate boundaries of the diauxic shift. (*a*) mtDNA copy number and cell size with time on the *x*-axis; (*b***)** the same data replotted as one series with time indicated by colour, showing that as the culture grows the association between mtDNA copy number and cell size is at first negative (*p* < 0.01), extending through and somewhat past the diauxic shift, and then positive thereafter (*p* < 10^−4^). Light-grey shading shows the confidence intervals for the slopes of the two linear regressions. (*c*) and (*d*) show ethanol and glucose concentration, and absorbance and density, respectively.

We found (figure 2) that the increase in mtDNA copy number increase begins at the very onset of the diauxic shift, as the rate of cellular proliferation slows and prior to total glucose exhaustion. The mtDNA copy number increase continues after the diauxic shift and appears to last throughout respiration. The rate of mtDNA copy number increase was faster during the diauxic shift and slower during the respiratory phase, though the difference in rate (0.15 h^−1^ versus 0.09 h^−1^) is not statistically significant in our data (electronic supplementary material, figure S8).

The portion of the increase in mtDNA copy number that occurs during the respiratory phase does not seem to require the diauxic shift to have previously occurred: we observed that cultures grown on a nonfermentable carbon source—glycerol alone or glycerol + ethanol—also underwent an mtDNA copy number increase (figure 1*c*). This increase is about half the total magnitude of the increase that occurred during growth on glucose, as the first measurement of mtDNA copy number (conducted at OD600 = 0.5, as in the glucose cultures) is about twice as high as during fermentative growth and the final measurements are indistinguishable between the two conditions.

### 2.4. mtDNA copy number changes and cell size

We estimated cell size by flow cytometry (figure 2*a* and *b*), supplemented with imaging via light microscopy (electronic supplementary material, figure S7). We observed a decline in median cell size, which carried through and for some time after the diauxic shift, before a later increase in cell size in the respiratory phase. This decline in cell volume is consistent with other observations in whole [35] or in part [34]. Slightly more than half of the mtDNA copy increase occurred while cell size was declining. Thus, there is an initially negative association between the rate of change of cell size and rate of change of mtDNA copy number, which eventually shifts to a positive association (figure 2*b*). The negative relationship between mtDNA copy number and cell size occurs over time across population averages; we note it is possible that at any given instant there is always a positive relationship within the population between cell size and mtDNA copy number, although we did not seek to confirm this.

### 2.5. Decrease in mtDNA copy number upon transfer from stationary phase

**Figure 3.**
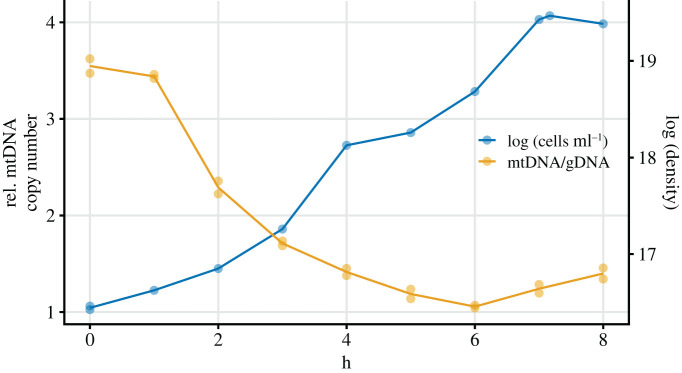
The drop in copy number immediately after transfer to fresh YPD. A saturated culture of the haploid laboratory strain was transferred, diluting 1/30, to fresh YPD. The relatively mild dilution permitted DNA extractions of small portions of the culture to begin immediately after transfer. Extractions for qPCR assays, and density and absorbance measurements, were performed at 1 h intervals for 8 h. From hours 0–3 the factor by which the mtDNA copy number fell and the population grew was similar, suggesting that there was little-to-no mtDNA production during this time.

Given that the mtDNA copy number increases during growth from dilute to saturated culture, we reasoned that the reverse process must take place: a decline in mtDNA copy number accompanies the resumption of growth that occurs upon transfer from a saturated culture to fresh media [36]. In order to observe this process, we transferred a relatively large number of late respiratory phase cells, sufficient that extractions could be immediately performed, to fresh YPD and began regular extractions immediately. The expected drop in mtDNA copy number occurred rapidly (figure 3) over the next 6 h.

During the first 3 h of growth (hours 0–3), the population increased by a factor of approximately 2.3 and the mtDNA copy number decreased by a factor of approximately 2.1, suggesting that virtually no mtDNA production was taking place during this time. During the next 3 h of growth (hours 3–6), the population increased by a factor of approximately 4.2 and the mtDNA copy number decreased by a factor of approximately 1.8, suggesting the resumption of mtDNA replication. Averaged over hours 1–6, the difference in Malthusian growth parameter between population growth rate and the mtDNA production rate is 0.25 h^−1^.

## 4. Discussion

The main result of this paper is that the mtDNA copy number is quite low during rapid fermentative growth and then rises dramatically during the course of typical batch culture growth on glucose, by a factor of up to approximately 4, depending on strain. While it was previously thought that the copy number does not change very much over different growth conditions [37], some degree of copy number change is not necessarily surprising. It has long been noted that the morphology of the yeast mitochondrial network is plastic and undergoes dramatic changes in response to different environmental conditions [22], and one previous study [19] reported an approximately 40% difference in mtDNA copy number between growth in aerobic and anaerobic conditions. The scale of the mtDNA copy number change that we report here, however, has not been previously reported.

We observe that the mtDNA copy number is stable during fermentation as long as glucose is abundant (figure 1*d* and electronic supplementary material, figure S6; note that for the experiment depicted in figure 2, sampling did not begin early enough to capture the period of stable copy number). The yeast transcriptome during fermentation is in a mostly steady state [38] until the diauxic shift nears; thus, it appears that this period of transcriptome stability is associated with copy number stability. While we find a fairly large strain-to-strain difference in mtDNA copy number (figure 1), this difference is minimized during fermentation, perhaps suggesting relatively tight control of a minimum complement of mitochondria during fermentation. We find that the absolute copy number, as measured by WGS, is 9 copies per haploid genome (figure 1*e*) during rapid fermentative growth. This is a lower copy number than is usually reported, perhaps because the copy number has not typically been assayed during rapid fermentative growth. We find that the absolute copy number varies over culture conditions to reach approximately 30–40 in haploids and just about twice that in diploids (a linear scaling with ploidy that has previously been reported [14,21,39], though see [20] for a counterexample).

The increase in mtDNA copy number begins before glucose has been fully exhausted (figure 2). This timing is very similar to the timing of the coordinated induction of stress and oxidative phosphorylation proteins that marks the earliest onset of the diauxic shift prior to complete glucose depletion [31–33]. All but one of the eight protein-coding genes on the *S. cerevisiae* mitochondrial genome are subunits of an oxidative phosphorylation enzyme complex. Thus, it would seem that the onset of the mtDNA copy number increase coincides with the onset of increased expression of most mitochondrially located genes. Data from Murphy *et al.* [32] that illustrates this point is re-plotted in electronic supplementary material, figure S9. This temporal correlation between expression levels and copy number is open to further investigation.

Although the mtDNA copy number increase is fairly rapid once it begins (approx. 0.15 units h^−1^; electronic supplementary material, figure S8), it is important to point out that our data do not suggest an absolute (i.e. per unit of time) increase in the rate of mtDNA replication upon the diauxic shift, because the rate of cellular reproduction also slows dramatically at this time. In fact, our data suggest that the rate of mtDNA production decreases at the diauxic shift, but that it decreases less than the rate of cellular reproduction does, so that there is a net increase in mtDNA production relative to each cell cycle. This is possible because in *S. cerevisiae*, mtDNA replication is decoupled from the cell cycle [36]. Proteomics data from Murphy *et al.* [32], replotted in electronic supplementary material, figure S6, shows a decrease in levels of *MIP1* (the sole mitochondrial polymerase) starting early in the diauxic shift, supporting the notion that the rate of mtDNA production actually slows in absolute terms as the copy number increases. *MIP1* was previously found to be responsible for changes in mtDNA copy number in starving cells [40]. We illustrate the point that relative rates of nuclear and mitochondrial genome growth determine the copy number with the toy model depicted in electronic supplementary material, figure S10, in which staggered alternation of equal genomic and mitochondrial growth rates leads to a cyclical rise and fall in the mtDNA copy number. Indeed, we observe that the mtDNA copy number drops sharply upon transfer to fresh media (figure 3): our data suggest that in such conditions, the lag for mitochondrial production to resume is longer than the lag for cellular reproduction to resume, causing a very rapid drop in mtDNA copy number until mtDNA production restarts.

The timing of the onset of the mtDNA copy number increase observed here also corresponds well with the timing of the mitochondrial volume increase reported by Bartolomeo *et al*. [34]. This increase in the absolute mitochondrial volume per cell begins early in the diauxic shift, just as the copy number increase does, and there is an initial doubling in mitochondrial volume in roughly the same timeframe as we observe a doubling in mtDNA copy number. The implication is that absolute mitochondrial volume per cell correlates well with mtDNA copy number per cell, though further work must be done to establish this.

The relationship between mtDNA copy number and cell size is more complex. The number of mtDNA nucleoids is correlated with the length of the mitochondrial network [41], which itself is correlated with cell volume [42]. This suggests that the mtDNA copy number is correlated with cell volume (with the caveat that the number of nucleoids and the mtDNA copy number are not necessarily correlated because the number of mtDNA genomes per nucleoid may vary). Indeed, it has been demonstrated [34] that at a single point in time during any phase of culture growth, cell volume and mtDNA copy number are positively associated within the population. We find, consistent with other reports [34,35], that cell volume decreases after the diauxic shift, and we observe that roughly the first half of the mtDNA copy number increase occurs while cell size is *decreasing* (figure 2*a,b*). Therefore, we find an initial negative association between the rate of change in copy number and the rate of change in cell volume, suggesting that change in cell size is not a causal driver of at least the initial portion of the mtDNA copy number increase. Subsequently, in late respiration, cell volume increases while the mtDNA copy number is still increasing, and the sign of the association become positive.

Interestingly, we observe a copy number increase during respiration even when there was never any fermentation (figure 1*c*). This suggests the possibility that there may be two separable phenomena: an approximate doubling of mtDNA copy number with the diauxic shift, and then a further rise in respiration that occurs whether or not there was a diauxic shift. The latter effect may also be related to the mtDNA copy number rise that has been reported when cells are transferred to starvation medium [40]. To the extent that either of these phenomena are adaptive, which is not necessarily the case, we might hypothesize that the initial and more rapid copy number increase during the diauxic shift reflects a demand for more mitochondria as their role transitions from fatty-acid production to primary energy generator, while the slower copy number increase that extends throughout respiration to stationary phase might reflect preparation for the sexual mode of reproduction of yeast, i.e. sporulation, which occurs in conditions of nitrogen starvation without a fermentable carbon source as well as under other types of nutrient deprivation [43]. During sporulation, the single genome doubling of meiosis is not accompanied by a corresponding increase in mtDNA copy number, and only a bit more than half of the total mtDNA of the parent diploid is included within one of the four haploid spores [44]. Thus, a sporulating diploid might require a high mtDNA copy number in order for each of its four haploid offspring to possess an adequate number of mtDNA copies. Indeed, the copy number dynamics of the wild diploid strain (figure 1*b*) are very different from the other strains. Unlike laboratory diploid strains, this wild diploid strain is a very ready sporulator and we observed that the great majority of cells had sporulated by the end of the experiment, as has been previously reported for some wild isolates grown on YPD [45]. In this strain, the mtDNA copy number actually falls after the initial doubling during the diauxic shift (figure 1*b*). Little remains known about the natural ecology of *S. cerevisiae* and its propensity to sporulate in natural environments [46].

## 5. Conclusion

In summary, we find that the mtDNA copy number of *S. cerevisiae* is quite low (9 per haploid genome) during the rapid growth of fermentation of glucose, and increases substantially during the diauxic shift and respiratory phases. One implication of this result is that future studies of the genetic control of mtDNA copy number should take care to compare the copy number at similar culture phases, in order to filter out apparent variation in copy number that might be caused by variation in growth dynamics.

We suggest that the fundamental facilitator of mtDNA copy number change is the fact that mtDNA production is decoupled from the cell cycle in *S. cerevisiae*. The absolute rate of mtDNA production per cell slows at the diauxic shift, but it slows less than cellular growth does so that the relative rate of mtDNA production increases and the copy number increases. An essentially reverse phenomenon occurs when stationary phase cells are transferred to fresh media. The molecular mechanisms responsible for linking the mtDNA production rate, however elastically, to the cellular growth rate at different points during the growth cycle remain largely open to further research. The increase in copy number begins very early in the diauxic shift when both transcriptional activity and mitochondrial volume are known to begin to change rapidly. In fact, the wholesale reorganization of yeast metabolism that begins with the onset of the diauxic shift may be initiated from the mitochondria [34]. The potential adaptive importance of mtDNA copy number change during this process is also open to further study.

## 6. Methods

### 6.1. Strains

yJHK112, a haploid, prototrophic, heterothallic, *MATa*, *BUD4*-corrected, and ymCherry-labelled W303 strain [47] was used as the haploid laboratory strain in this paper. We created the diploid laboratory strain from this strain by transforming [48] the haploid laboratory strain with plasmid pRY003, temporarily providing a functional *HO* locus allowing mating-type switching and subsequent mating. pRY003 was a gift from John McCusker (Addgene plasmid no. 81043; http://n2t.net/addgene:81043; RRID: Addgene_81043). The ploidy of the resulting strain was confirmed by: (1) the ability to produce tetrads after plating to sporulation media; (2) flow cytometry for total genomic content; and (3) the presence of a PCR product for both the *MATa* and *MATα* loci. YPS623, the wild diploid strain used in this paper, was isolated from Mettler's Woods, NJ, following procedures described in [49]. YPS 2066, the haploid wild strain used in this paper, originated from an ascospore of YPS623, isolated by microdissection, and subsequently made heterothallic by knocking out the *HO* locus by standard genetic techniques.

### 6.2. Growth conditions

Growth was carried out in liquid media in Erlenmeyer flasks kept at 30°C and shaken at 200 rpm. For all experiments, an initial growth cycle was carried out in 6 ml media in a 50 ml flask upon revival from frozen storage. Unless otherwise noted, all experiments described in this paper were carried out in 50 ml media in a 250 ml flask. Except for the experiment described in figure 3, all transfers to start experiments were 1:2000 (25 µl into 50 ml) dilutions from pregrown cultures. Three kinds of media were used: YPD (also known as YEPD) (2% peptone, 2% glucose, 1% yeast extract); YPG (2% peptone, 3% glycerol, 1% yeast extract); and YPEG (2% peptone, 2.6% glycerol, 2.6% ethanol, 1% yeast extract).

### 6.3. Measurement of culture properties

Density measurements were performed by manually counting the number of cells, via microscope, in an improved Neubauer-style haemocytometer. Depending on culture density, dilutions of up to 1/200 (in water) were performed before counting. Still-attached buds were counted as a separate cell if they had a diameter greater than half that of the mother.

Absorbance was measured at 600 nm (OD600) by standard methods. If the OD600 was greater than 1, the culture was diluted 1/10 in the appropriate media in order to obtain a reading within the better part of the dynamic range of the spectrophotometer. Using a data set of dual (diluted and undiluted) measurements to interpolate, all diluted OD600 measurements were converted to the scale of undiluted measurements so that only one scale of measurement is presented.

To measure glucose and ethanol concentrations, 0.5 ml culture samples were spun down and the supernatant stored at −20°C. Samples were then defrosted for glucose and ethanol measurement. R-Biopharm kit 10 176 290 035 and Megazyme kit K-GLUHK-110A were used to measure ethanol and glucose concentration, respectively. Both kits were used according to the manufacturer's instructions.

### 6.4. DNA extractions

DNA extractions were performed using Zymo Quick-DNA Fungal/Bacterial kits (D6005), which employ glass beads to physically lyse cells followed by a spin column-based purification. In order to limit any effects of different extraction conditions, and because extractions were performed from growing cultures at different time points and hence different densities, within a single experiment the extraction volume was adjusted so that the absolute number of cells extracted was similar for each timepoint. As an exception to this practice, extractions destined for whole-genome sequencing were greater in cell number than extractions destined for qPCR only.

In early exploratory experiments, similar results as those described here were obtained with both purely enzymatic extraction protocols (Zymo D2002) and a non-kit-based glass beads/phenol:chloroform extraction protocol.

### 6.5. qPCR

qPCR assays for mtDNA content were performed using the relative standard curve method. For both laboratory and wild strains, a single large-scale extraction from the haploid strain was performed when the culture was in fermentation, at absorbance OD600 = 0.6. For every qPCR run, a fivefold serial dilution of this single extract served as the standard curve. Each sample was run with two sets of primers, identical to the primers employed in Puddu *et al*. [21]. We employed one set of primers to target a region of nDNA and another to amplify a region of mtDNA. The nDNA primer set (fwd: TGCTTTGTCAAATGGATCATATGG, rev: CCTGGAACCAAGTGAACAGTACAA) targeted a short region of GAL1 (chr II: 280 382–280 459), and the mtDNA primer set (fwd: CACCACTAATTGAAAACCTGTCTG, rev: GATTTATCGTATGCTCATTTCCAA) targeted a short region of COX1 in the mtDNA (25 574–25 686). For each sample, the quantity of mtDNA and nDNA relative to the standard curve was computed, and the ratio of the two was recorded as the relative mtDNA copy number. Threshold calculations, curve fitting, and quantity calculations were performed by QuantStudio 3 software. All wells were run in triplicate per run and outliers from each triplet were occasionally removed manually. Each qPCR run was performed on a 96-well plate allowing a maximum of 11 unknown samples to be assayed for relative mtDNA copy number (11 samples × 2 primer sets × in triplicate = 66 wells, plus 5 standards × 2 primer sets × in triplicate = 30 wells, for a total of 96 wells).

### 6.6. Sequencing and copy number analysis

The mtDNA copy number analysis was conducted similarly to Puddu *et al*. [21] and carried out working from short-read (Illumina) WGS data as follows (also see sequencing_pipeline.txt in the online data). Reads were first processed by TrimGalore with options –nextera –stringency 3 –paired –quality 20 –length 50. Reads were mapped to the S288C R64 reference genome by bwa. Duplicate reads were removed by biobambam2's bammarkduplicates and then read depth was computed by samtools depth. The mtDNA genome includes many regions of high AT content that are not covered well; hence, the mtDNA copy number was computed as the median read depth in a region of the mtDNA genome with regular coverage (COX1 from 14 000–20 000; see electronic supplementary material, figure S3) divided by the median read depth in the nuclear genome. The mtDNA copy number calculated using COX3 instead (79 213–80 022) was very similar. As controls, we calculated copy numbers for other repetitive elements (electronic supplementary material, figure S2), which did not change over the course of culture growth.

Data presented in figure 1*e* and electronic supplementary material, figure S1, were produced from short-read sequencing. Initially, we suspected that the coverage from short-read sequencing would be too irregular to permit accurate copy number estimates. Therefore, in preliminary experiments, we compared short-read (Illumina) and long-read (Nanopore) sequencing from the same samples. While the coverage for the Nanopore data was indeed somewhat smoother (electronic supplementary material, figures S3 and S4), the mitochondrial coverage for the short-read data was acceptably regular. Indeed, the two methods gave near-identical results (electronic supplementary material, table S5).

### 6.7. Cell size

We used forward scatter (FSC) as a proxy for cell size [50]. Culture samples of varying volume (to account for changing culture density) were fixed in 70% ethanol and stored at 4°C, then spun down, washed once, and resuspended in sodium citrate buffer. After a 1/10 dilution, samples were sonicated twice for 10 s at 30% power, examined microscopically to ensure sonication was effective, and then diluted 1/20 for flow cytometry on a Guava EasyCyte. Samples were run for 90 s at a flow rate of Very Low, with doublet discrimination performed by removing off-diagonal points on FSC-W versus FSC-H.

We obtained an independent set of measurements of cell size by light microscopy. Live cells were imaged on a Nexcelom Cellometer X2 and processed using object detection and measurement pipeline (available in the online supplement) implemented in CellProfiler [51]. In this pipeline, all processed images were reviewed and any cell aggregations that were not declustered properly were manually removed from the output (sample images included in electronic supplementary material, figure S7).

## Data Availability

All code to generate figures in the submitted manuscript, along with the underlying data, is available for review here: https://datadryad.org/stash/share/EVqfjBsUcDtCQ_9T7_H8LGkXJntOeBzoZ0X8Er86gl0 [52]. Electronic supplementary material is available online [53].
